# Supplementary material for: Gender Differences in Psychological Symptoms and Quality of Life in Patients with Inflammatory Bowel Disease in China: A Multicenter Study
Source: J Clin Med. 2023 Feb 23;12(5):1791. doi: 10.3390/jcm12051791 (PMC10002859; doi:10.3390/jcm12051791)
Supplement: Supplementary file 1 [file jcm-12-01791-s001.zip › Supplementary Table S1.pdf]

Supplementary Table S1. Scores of psychological symptoms, sleep quality and quality of life related scales in IBD patients of different genders（score，$\bar{x}\pm s$）

Projects	IBD(n=2478)		t	P-value	UC(n=1371)		t	P-value	CD(n=1107)		t	P-value
	Male (n=1547)	Female (n=931)			Male (n=754)	Female (n=617)			Male (n=793)	Female (n=314)		
GAD-7 score	6.17±4.97	7.40±5.57	-5.548	<0.001	6.23±5.17	7.65±5.72	-4.772	<0.001	6.12±4.78	6.92±5.26	-2.460	0.014
PHQ-9 score	6.70±5.81	7.59±6.52	-3.407	0.001	6.53±5.89	7.69±6.63	-3.404	0.001	6.87±5.74	7.39±6.31	-1.305	0.192
PSQI score	6.69±3.59	7.24±4.01	-3.422	0.001	6.80±3.61	7.25±4.03	-2.150	0.032	6.59±3.57	7.23±3.99	-2.456	0.014
IBD-Q score	177.51±30.12	173.00±32.24	3.455	0.001	173.80±32.21	170.78±32.97	1.710	0.088	181.04±27.55	177.37±30.34	1.860	0.063
Bowel symptoms	58.05±9.92	56.79±10.61	2.920	0.004	56.16±10.76	55.63±10.92	0.902	0.367	59.85±8.69	59.09±9.59	1.218	0.224
Systemic symptoms	27.25±5.31	26.67±5.57	2.567	0.010	26.96±5.54	26.38±5.65	1.908	0.057	27.52±5.07	27.24±5.36	0.814	0.416
Emotional ability	66.93±12.25	64.50±13.46	4.501	<0.001	65.70±12.70	63.77±13.60	2.714	0.007	68.10±11.71	65.93±13.08	2.558	0.011
Social ability	25.29±4.93	25.04±5.18	1.175	0.240	24.98±5.21	25.00±5.23	-0.069	0.945	25.58±4.64	25.11±5.10	1.396	0.163
