# Supplementary material for: Gender Differences in Psychological Symptoms and Quality of Life in Patients with Inflammatory Bowel Disease in China: A Multicenter Study
Source: J Clin Med. 2023 Feb 23;12(5):1791. doi: 10.3390/jcm12051791 (PMC10002859; doi:10.3390/jcm12051791)
Supplement: Supplementary file 1 [file jcm-12-01791-s001.zip › Supplementary Table S2.pdf]

Supplementary Table S2. Univariate analysis of influencing factors of psychological symptoms, sleep quality and quality of life in male patients with IBD [n(%)]

Variable	Anxiety			Depression			Sleep disturbance			Poor quality of life		
	No (*n*=1200)	Yes (*n*=347)	*P*-value	No (*n*=623)	Yes (*n*=308)	*P*-value	No (*n*=644)	Yes (*n*=903)	*P*-value	No (*n*=1003)	Yes (*n*=544)	*P*-value
Age (years old)			0.282			0.556			0.725			0.895
18～35	651(54.3)	181(52.2)		599(53.6)	233 (54.3)		352(54.7)	480(53.2)		543(54.1)	289(53.1)	
36～60	473(39.4)	150(43.2)		448(40.1)	175(40.8)		252(39.1)	371(41.1)		402(40.1)	221(40.6)	
＞60	76(6.3)	16(4.6)		71(6.4)	21(4.9)		40(6.2)	52(5.8)		58(5.8)	34(6.3)	
First visit			0.004			<0.001			0.029			<0.001
Yes	212(17.7)	85(24.5)		189(16.9)	108(25.2)		107(16.6)	190(21.0)		153(15.3)	144(26.5)	
No	988(82.3)	262(75.5)		929(83.1)	321(74.8)		537(83.4)	713(79.0)		850(84.7)	400(73.5)	
Disease activity			<0.001			0.002			0.077			<0.001
Remission	518(43.2)	107(30.8)		478(42.8)	147(34.3)		277(43.0)	348(38.5)		519(51.7)	106(19.5)	
Active	682(56.8)	240(69.2)	0.159	640(57.2)	282(65.7)	0.371	367(57.0)	555(61.5)	0.251	484(48.3)	438(80.5)	<0.001
Mild	274(40.2)	94(39.2)		261(40.8)	107(37.9)		151(41.1)	217(39.1)		234 (48.3)	134(30.6)	
Moderate	317(46.5)	102(42.5)		292(45.6)	127(45.0)		171(46.6)	248(44.7)		204(42.1)	215(49.1)	
Severe	91(13.3)	44(18.3)		87(13.6)	48(17.0)		45 (12.3)	90(16.2)		46(9.5)	89(20.3)	
Disease type			0.015			0.311			0.827			<0.001
UC	565(47.1)	189(54.5)		536(47.9)	218(50.8)		316(49.1)	438(48.5)		454(45.3)	300(55.1)	
CD	635(52.9)	158(45.5)		582(52.1)	211(49.2)		328(50.9)	465(51.5)		549(54.7)	244(44.9)	
Disease duration			0.634			0.087			0.087			0.015
<2 years	438(36.5)	122(35.3)		423(37.9)	137(32.0)		253(39.3)	307(34.0)		337(33.7)	223(41.0)	
2～5 years	390(32.5)	122(35.3)		357(32.0)	155(36.2)		207(32.2)	305(33.8)		349(34.9)	163(30.0)	
>5 years	371(30.9)	102(29.5)		337(30.2)	136(31.8)		183(28.5)	290(32.2)		315(31.5)	158(29.0)	

Diarrhea			0.632			0.440			0.890			<0.001
Yes	716(59.7)	212(61.1)		664(59.4)	264(61.5)		385(59.8)	543(60.1)		556(55.4)	372(68.4)	
No	484(40.3)	135(38.9)		454(40.6)	165(38.5)		259(40.2)	360(39.9)		447(44.6)	172(31.6)	
Hematochezia			<0.001			0.079			0.101			<0.001
Yes	443(36.9)	166(47.8)		425(38.0)	184(42.9)		238(37.0)	371(41.1)		325(32.4)	284(52.2)	
No	757(63.1)	181(52.2)		693(62.0)	245(57.1)		406(63.0)	532(58.9)		678(67.6)	260(47.8)	
Abdominal pain			0.221			0.010			0.931			<0.001
Yes	668(55.7)	206(59.4)		609(54.5)	265(61.8)		363(56.4)	511(56.6)		511(50.9)	363(66.7)	
No	532(44.3)	141(40.6)		509(45.5)	164(38.2)		281(43.6)	392(43.4)		492(49.1)	181(33.3)	
Extraintestinal manifestation			0.184			0.249			0.157			0.014
Yes	88(7.3)	33(9.5)		82(7.3)	39(9.1)		43(6.7)	78(8.6)		66(6.6)	55(10.1)	
No	1112(92.7)	314(90.5)		1036(92.7)	390(90.9)		601(93.3)	825(91.4)		937(93.4)	489(89.9)	
Comorbidities			0.958			0.625			0.308			0.181
Yes	113(9.4)	33(9.5)		103(9.2)	43(10.0)		55(8.5)	91(10.1)		102(10.2)	44(8.1)	
No	1087(90.6)	314(90.5)		1015(90.8)	386(90.0)		589(91.5)	812(89.9)		901(89.8)	500(91.9)	
5-Aminosalicylic acid			0.045			0.504			0.429			-
Yes	678(56.5)	217(62.5)		641(57.3)	254(59.2)		365(56.7)	530(58.7)		-	-	
No	522(43.5)	130(37.5)		477(42.7)	175(40.8)		279(43.3)	373(41.3)		-	-	
Glucocorticoids			0.726			0.380			0.543			-
Yes	181(15.1)	55(15.9)		165(14.8)	71(16.6)		94(14.6)	142(15.7)		-	-	
No	1019(84.9)	292(84.1)		953(85.2)	358(83.4)		550(85.4)	761(84.3)		-	-	
Immunosuppressants			0.077			0.972			0.551			0.138
Yes	203(16.9)	45(13.0)		179(16.0)	69(16.1)		99(15.4)	149(16.5)		171(17.0)	77(14.2)	
No	997(83.1)	302(87.0)		939(84.0)	360(83.9)		545(84.6)	754(83.5)		832(83.0)	467(85.8)	
Biological agents			0.128			0.526			0.966			0.154

Yes	688(57.3)	183(52.7)		635(56.8)	236(55.0)		363(56.4)	508(56.3)		578(57.6)	293(53.9)	
No	512(42.7)	164(47.3)		483(43.2)	193(45.0)		281(43.6)	395(43.7)		425(42.4)	251(46.1)	
IBD-related surgery			0.135			0.237			0.768			0.572
Yes	180(15.0)	41(11.8)		167(14.9)	54(12.6)		90(14.0)	131(14.5)		147(14.7)	74(13.6)	
No	1020(85.0)	306(88.2)		951(85.1)	375(87.4)		554(86.0)	772(85.5)		856(85.3)	470(86.4)	
Anxiety			-			<0.001			<0.001			<0.001
Yes	-	-		79(7.1)	268(62.5)		42(6.5)	305(33.8)		154(15.4)	193(35.5)	
No	-	-		1039(92.9)	161(37.5)		602(93.5)	598(66.2)		849(84.6)	351(64.5)	
Depression			<0.001			-			<0.001			<0.001
Yes	161(13.4)	268(77.2)		-	-		37(5.7)	392(43.4)		195(19.4)	234(43.0)	
No	1039(86.6)	79(22.8)		-	-		607(94.3)	511(56.6)		808(80.6)	310(57.0)	
Sleep disturbance			<0.001			<0.001			-			<0.001
Yes	598(49.8)	305(87.9)		511(45.7)	392(91.4)		-	-		513(51.1)	390(71.7)	
No	602(50.2)	42(12.1)		607(54.3)	37(8.6)		-	-		490(48.9)	154(28.3)	
Poor quality of life			<0.001			<0.001			<0.001			-
Yes	351(29.3)	193(55.6)		310(27.7)	234(54.5)		154(23.9)	390(43.2)		-	-	
No	849(70.8)	154(44.4)		808(72.3)	195(45.5)		490(76.1)	513(56.8)		-	-	
