# Supplementary material for: Gender Differences in Psychological Symptoms and Quality of Life in Patients with Inflammatory Bowel Disease in China: A Multicenter Study
Source: J Clin Med. 2023 Feb 23;12(5):1791. doi: 10.3390/jcm12051791 (PMC10002859; doi:10.3390/jcm12051791)
Supplement: Supplementary file 1 [file jcm-12-01791-s001.zip › Supplementary Table S3.pdf]

Supplementary Table S3. Univariate analysis of influencing factors of psychological symptoms, sleep quality and quality of life in female patients with IBD [n(%)]

Variable	Anxiety			Depression			Sleep disturbance			Poor quality of life		
	No (*n*=647)	Yes (*n*=284)	*P*-value	No (*n*=623)	Yes (*n*=308)	*P*-value	No (*n*=343)	Yes (*n*=588)	*P*-value	No (*n*=542)	Yes (*n*=389)	*P*-value
Age (years old)			0.969			0.939			0.176			0.652
18～35	288(44.5)	124(43.7)		276(44.3)	136(44.2)		165(48.1)	247(42.0)		246(45.4)	166(42.7)	
36～60	326(50.4)	145(51.1)		316(50.7)	155(50.3)		163(47.5)	308(52.4)		270(49.8)	201(51.7)	
＞60	33(5.1)	15(5.3)		31(5.0)	17(5.5)		15(4.4)	33(5.6)		26(4.8)	22(5.7)	
First visit			0.050			0.083			0.301			<0.001
Yes	125(19.3)	71(25.0)		121(19.4)	75(24.4)		66(19.2)	130(22.1)		85(15.7)	111(28.5)	
No	522(80.7)	213(75.0)		502(80.6)	233(75.6)		277(80.8)	458(77.9)		457(84.3)	278(71.5)	
Disease activity			<0.001			0.001			0.017			<0.001
Remission	247(38.2)	74(26.1)		237(38.0)	84(27.3)		135(39.4)	186(31.6)		262(48.3)	59(15.2)	
Active	400(61.8)	210(73.9)	0.030	386(62.0)	224(72.7)	0.004	208(60.6)	402(68.4)	0.058	280(51.7)	330(84.8)	<0.001
Mild	148(37.0)	72(34.3)		135(35.0)	85(37.9)		74(35.6)	146(36.3)		119(42.5)	101(30.6)	
Moderate	213(53.3)	102(48.6)		215(55.7)	100(44.6)		117(56.3)	198(49.3)		146(52.1)	169(51.2)	
Severe	39(9.8)	36(17.1)		36(9.3)	39(17.4)		17(8.2)	58(14.4)		15(5.4)	60(18.2)	
Disease type			0.076			0.246			0.850			0.005
UC	417(64.5)	200(70.4)		405(65.0)	212(68.8)		226(65.9)	391(66.5)		339(62.5)	278(71.5)	
CD	230(35.5)	84(29.6)		218(35.0)	96(31.2)		117(34.1)	197(33.5)		203(37.5)	111(28.5)	
Disease duration			0.641			0.753			0.481			0.228
<2 years	269(41.6)	110(38.7)		255(40.9)	124(40.3)		148(43.1)	231(39.3)		209(38.6)	170(43.7)	
2～5 years	170(26.3)	82(28.9)		164(26.3)	88(28.6)		91(26.5)	161(27.4)		156(28.8)	96(24.7)	
>5 years	208(32.1)	92(32.4)		204(32.7)	96(31.2)		104(30.3)	196(33.3)		177(32.7)	123(31.6)	

Diarrhea			0.094			0.041			0.672			<0.001
Yes	398(61.5)	191(67.3)		380(61.0)	209(67.9)		220(64.1)	369(62.8)		296(54.6)	293(75.3)	
No	135(42.7)	93(32.7)		243(39.0)	99(32.1)		123(35.9)	219(37.2)		246(45.4)	96(24.7)	
Hematochezia			0.002			0.031			0.200			<0.001
Yes	290(44.8)	159(56.0)		285(45.7)	164(53.2)		156(45.5)	293(49.8)		212(39.1)	237(60.9)	
No	357(55.2)	125(44.0)		338(54.3)	144(46.8)		187(54.5)	295(50.2)		330(60.9)	152(39.1)	
Abdominal pain			0.330			0.810			0.425			<0.001
Yes	372(57.5)	173(60.9)		363(58.3)	182(59.1)		195(56.9)	350(59.5)		282(52.0)	263(67.6)	
No	275(42.5)	111(39.1)		260(41.7)	126(40.9)		148(43.1)	238(40.5)		260(48.0)	126(32.4)	
Extraintestinal manifestation			0.156			0.905			0.928			0.967
Yes	59(9.1)	18(6.3)		52(8.3)	25(8.1)		28(8.2)	49(8.3)		45(8.3)	32(8.2)	
No	588(90.9)	266(93.7)		571(91.7)	283(91.9)		315(91.8)	539(91.7)		497(91.7)	357(91.8)	
Comorbidities			0.559			0.115			0.725			0.469
Yes	43(6.6)	16(5.6)		45(7.2)	14(4.5)		23(6.7)	36(6.1)		37(6.8)	22(5.7)	
No	604(93.4)	268(94.4)		578(92.8)	294(95.5)		320(93.3)	552(93.9)		505(93.2)	367(94.3)	
5-Aminosalicylic acid			0.032			0.324			0.285			-
Yes	428(66.2)	208(73.2)		419(67.3)	217(70.5)		227(66.2)	409(69.6)		-	-	
No	219(33.8)	76(26.8)		204(32.7)	91(29.5)		116(33.8)	179(30.4)		-	-	
Glucocorticoids			0.538			0.520			0.367			-
Yes	99(15.3)	48(16.9)		95(15.2)	52(16.9)		59(17.2)	88(15.0)		-	-	
No	548(84.7)	236(83.1)		528(84.8)	256(83.1)		284(82.8)	500(85.0)		-	-	
Immunosuppressants			0.015			0.085			0.159			0.568
Yes	89(13.8)	23(8.1)		83(13.3)	29(9.4)		48(14.0)	64(10.9)		68(12.5)	44(11.3)	
No	558(86.2)	261(91.9)		540(86.7)	279(90.6)		295(86.0)	524(89.1)		474(87.5)	345(88.7)	
Biological agents			0.036			0.325			0.135			0.023

Yes	294(45.4)	108(38.0)		276(44.3)	126(40.9)		159(46.4)	243(41.3)		251(46.3)	151(38.8)	
No	353(54.6)	176(62.0)		347(55.7)	182(59.1)		184(53.6)	345(58.7)		291(53.7)	238(61.2)	
IBD-related surgery			0.114			0.276			0.564			0.013
Yes	64(9.9)	19(6.7)		60(9.6)	23(7.5)		33(9.6)	50(8.5)		59(10.9)	24(6.2)	
No	583(90.1)	265(93.3)		563(90.4)	285(92.5)		310(90.4)	538(91.5)		483(89.1)	365(93.8)	
Anxiety			-			<0.001			<0.001			<0.001
Yes	-	-		52(8.3)	232(75.3)		34(9.9)	250(42.5)		117(21.6)	167(42.9)	
No	-	-		571(91.7)	76(24.7)		309(90.1)	338(57.5)		425(78.4)	222(57.1)	
Depression			<0.001			-			<0.001			<0.001
Yes	76(11.7)	232(81.7)		-	-		32(9.3)	276(46.9)		129(23.8)	179(46.0)	
No	571(88.3)	52(18.3)		-	-		311(90.7)	312(53.1)		413(76.2)	210(54.0)	
Sleep disturbance			<0.001			<0.001			-			<0.001
Yes	338(52.2)	250(88.0)		312(50.1)	276(89.6)		-	-		309(57.0)	279(71.7)	
No	309(47.8)	34(12.0)		311(49.9)	32(10.4)		-	-		233(43.0)	110(28.3)	
Poor quality of life			<0.001			<0.001			<0.001			-
Yes	222(34.3)	167(58.8)		210(33.7)	179(58.1)		110(32.1)	279(47.4)		-	-	
No	425(65.7)	117(41.2)		413(66.3)	129(41.9)		233(67.9)	309(52.6)		-	-	
