# Supplementary material for: Gender Differences in Psychological Symptoms and Quality of Life in Patients with Inflammatory Bowel Disease in China: A Multicenter Study
Source: J Clin Med. 2023 Feb 23;12(5):1791. doi: 10.3390/jcm12051791 (PMC10002859; doi:10.3390/jcm12051791)
Supplement: Supplementary file 1 [file jcm-12-01791-s001.zip › Supplementary Table S4.pdf]

Supplementary Table S4. Multivariate Logistic analysis of influencing factors of psychological symptoms, sleep quality and quality of life in male patients with IBD

Variable	*β*	*S.E.*	*Wald*χ^2	*P*-value	*OR*	95% CI	
						Lower limit	Upper limit
**Anxiety**							
First visit (Yes vs No)	-0.008	0.192	0.002	0.966	0.992	0.681	1.445
Disease activity (Active vs Remission)	0.182	0.185	0.975	0.323	1.200	0.836	1.723
Disease type (CD vs UC)	-0.120	0.211	0.326	0.568	0.887	0.587	1.340
Hematochezia (Yes vs No)	0.265	0.196	1.837	0.175	1.304	0.888	1.913
5-Aminosalicylic acid (Yes vs No)	0.017	0.196	0.007	0.931	1.017	0.692	1.494
Depression (Yes vs No)	2.697	0.167	262.123	0.000	14.834	10.702	20.562
Sleep disturbance (Yes vs No)	0.871	0.204	18.290	0.000	2.388	1.603	3.559
Poor quality of life (Yes vs No)	0.444	0.165	7.215	0.007	1.558	1.127	2.154
**Depression**							
First visit (Yes vs No)	0.347	0.185	3.508	0.061	1.414	0.984	2.033
Disease activity (Active vs Remission)	-0.201	0.169	1.411	0.235	0.818	0.587	1.140
Abdominal pain (Yes vs No)	0.328	0.157	4.362	0.037	1.388	1.020	1.887
Anxiety (Yes vs No)	2.695	0.166	263.261	0.000	14.806	10.692	20.503
Sleep disturbance (Yes vs No)	2.058	0.201	104.480	0.000	7.832	5.278	11.622
Poor quality of life (Yes vs No)	0.636	0.164	15.092	0.000	1.888	1.370	2.602
**Sleep disturbance**							
First visit (Yes vs No)	0.029	0.152	0.036	0.850	1.029	0.764	1.386
Anxiety (Yes vs No)	0.847	0.203	17.354	0.000	2.332	1.566	3.473
Depression (Yes vs No)	2.035	0.200	103.125	0.000	7.649	5.165	11.327

Poor quality of life (Yes vs No)	0.483	0.128	14.298	0.000	1.622	1.262	2.084
**Poor quality of life**							
First visit (Yes vs No)	0.473	0.152	9.669	0.002	1.604	1.191	2.161
Disease activity (High vs Low)	0.665	0.065	103.925	0.000	1.944	1.711	2.209
Disease type (CD vs UC)	-0.019	0.146	0.017	0.895	0.981	0.737	1.306
Disease course (Long vs Short)	-0.107	0.075	2.050	0.152	0.898	0.776	1.040
Diarrhea (Yes vs No)	0.250	0.131	3.663	0.056	1.284	0.994	1.658
Hematochezia (Yes vs No)	0.223	0.149	2.227	0.136	1.250	0.932	1.675
Abdominal pain (Yes vs No)	0.423	0.128	10.949	0.001	1.526	1.188	1.961
Extraintestinal manifestation (Yes vs No)	0.303	0.218	1.932	0.165	1.355	0.883	2.078
Anxiety (Yes vs No)	0.460	0.169	7.378	0.007	1.584	1.137	2.208
Depression (Yes vs No)	0.644	0.165	15.175	0.000	1.905	1.377	2.634
Sleep disturbance (Yes vs No)	0.547	0.137	15.941	0.000	1.728	1.321	2.260
