# Supplementary material for: Gender Differences in Psychological Symptoms and Quality of Life in Patients with Inflammatory Bowel Disease in China: A Multicenter Study
Source: J Clin Med. 2023 Feb 23;12(5):1791. doi: 10.3390/jcm12051791 (PMC10002859; doi:10.3390/jcm12051791)
Supplement: Supplementary file 1 [file jcm-12-01791-s001.zip › Supplementary Table S5.pdf]

Supplementary Table S5. Multivariate Logistic analysis of influencing factors of psychological symptoms, sleep quality and quality of life in female patients with IBD

Variable	*β*	*S.E.*	*Wald* χ^2	*P*-value	*OR*	95% CI	
						Lower limit	Upper limit
**Anxiety**							
First visit (Yes vs No)	0.066	0.243	0.074	0.786	1.068	0.664	1.718
Disease activity (High vs Low)	0.138	0.118	1.373	0.241	1.148	0.911	1.447
Hematochezia (Yes vs No)	0.189	0.227	0.691	0.406	1.208	0.774	1.886
5-Aminosalicylic acid (Yes vs No)	0.056	0.286	0.038	0.845	1.058	0.604	1.853
Immunosuppressants (Yes vs No)	-0.476	0.338	1.978	0.160	0.621	0.320	1.206
Biological agents (Yes vs No)	-0.255	0.262	0.952	0.329	0.775	0.464	1.294
Depression (Yes vs No)	3.204	0.207	240.438	0.000	24.637	16.432	36.938
Sleep disturbance (Yes vs No)	0.890	0.249	12.797	0.000	2.435	1.495	3.964
Poor quality of life (Yes vs No)	0.379	0.216	3.082	0.079	1.461	0.957	2.232
**Depression**							
Disease activity (High vs Low)	-0.054	0.116	0.218	0.641	0.947	0.755	1.189
Diarrhea (Yes vs No)	0.262	0.216	1.467	0.226	1.299	0.851	1.984
Hematochezia (Yes vs No)	-0.073	0.220	0.110	0.740	0.929	0.604	1.431
Anxiety (Yes vs No)	3.192	0.205	241.732	0.000	24.343	16.278	36.404
Sleep disturbance (Yes vs No)	1.588	0.246	41.752	0.000	4.892	3.022	7.918
Poor quality of life (Yes vs No)	0.566	0.212	7.117	0.008	1.761	1.162	2.670
**Sleep disturbance**							
Disease activity (Active vs Remission)	0.068	0.164	0.170	0.680	1.070	0.776	1.477
Anxiety (Yes vs No)	0.883	0.245	13.011	0.000	2.418	1.497	3.907

Depression (Yes vs No)	1.561	0.244	41.032	0.000	4.766	2.956	7.685
Poor quality of life (Yes vs No)	0.251	0.167	2.272	0.132	1.285	0.927	1.781
**Poor quality of life**							
First visit (Yes vs No)	0.543	0.185	8.572	0.003	1.721	1.197	2.475
Disease activity (High vs Low)	0.716	0.090	63.217	0.000	2.046	1.715	2.441
Disease type (CD vs UC)	0.168	0.211	0.631	0.427	1.183	0.782	1.789
Diarrhea (Yes vs No)	0.636	0.166	14.626	0.000	1.888	1.363	2.615
Hematochezia (Yes vs No)	0.165	0.178	0.859	0.354	1.180	0.832	1.674
Abdominal pain (Yes vs No)	0.413	0.162	6.522	0.011	1.511	1.101	2.073
Biological agents (Yes vs No)	-0.492	0.183	7.254	0.007	0.611	0.427	0.875
IBD-related surgery (Yes vs No)	-0.024	0.302	0.006	0.936	0.976	0.540	1.766
Anxiety (Yes vs No)	0.359	0.219	2.693	0.101	1.431	0.933	2.197
Depression (Yes vs No)	0.618	0.219	7.953	0.005	1.855	1.207	2.851
Sleep disturbance (Yes vs No)	0.271	0.172	2.480	0.115	1.312	0.936	1.838
